# Poxviruses in dogs and cats: a genuine threat?

**DOI:** 10.29374/2527-2179.bjvm008724

**Published:** 2025-03-14

**Authors:** Aline Santana da Hora, Isabelle Ezequiel Pedrosa, Lana Isabella Gila

**Affiliations:** 1 Laboratório de Investigação Etiológica Veterinária (LIvE Vet), Faculdade de Medicina Veterinária, Universidade Federal de Uberlândia, Uberlândia, MG, Brazil.; 2 Programa de Pós-Graduação em Ciências Veterinárias (PPGCVET), Universidade Federal de Uberlândia, Uberlândia, MG, Brazil.

**Keywords:** ***:*** monkeypox virus, vaccinia virus, Brazilian porcupinepox virus, Poxviridae, vírus da varíola dos macacos, vaccinia vírus, Brazilian porcupinepox virus, Poxviridae

## Abstract

During the global Mpox outbreak, the potential role of dogs and cats in the epidemiological chain of monkeypox virus was suspected. Additionally, Brazil is endemic for vaccinia virus and the recently described Brazilian porcupinepox virus, both of which require further investigation regarding their potential reservoirs. Given that dogs and cats are among the most popular pets worldwide and that poxvirus infections could impact multispecies households, this study aimed to assess the circulation of poxviruses in pets during the Mpox pandemic. A pan-pox polymerase chain reaction assay was applied to blood samples from 608 dogs and 271 cats. Despite the potential for detecting poxviruses in companion animals, no molecular evidence of poxvirus infection was found in the studied population.

## Resumo

Durante o surto global de Mpox, suspeitou-se da possível participação de cães e gatos na cadeia epidemiológica do monkeypox. Além disso, o Brasil é endêmico para o vaccinia vírus poxvírus vaccínia virus e o recentemente descrito Brazilian porcupinepox virus, os quais demandam estudos mais aprofundados sobre seus possíveis reservatórios. Considerando que esses animais estão entre os pets mais populares do mundo e o impacto potencial das infecções por poxvírus em famílias multiespécies, este estudo teve como objetivo avaliar a circulação de poxvírus em animais de estimação durante a pandemia de Mpox. Foi realizada a reação em cadeia pela polimerase pan-pox em amostras de sangue de 608 cães e 271 gatos. Apesar da possibilidade de detecção de algumas espécies de poxvírus em animais de companhia, não foi observado material genético de poxvírus na população estudada.

Poxviruses, belonging to the *Poxviridae* family, are widely recognized and highly concerning, with some exhibiting zoonotic potential—particularly members of the *Orthopoxvirus* genus (Oliveira et al., 2017). Between January 2022 and August 2024, *Orthopoxvirus monkeypox* (monkeypox virus [MPV]) has caused human infections in 123 countries, resulting in 106,310 confirmed cases and 234 deaths. Brazil ranks second globally in confirmed human MPV cases, with 12,206 reported (World Health Organization, 2024). MPV is a zoonotic virus with well-documented hosts, including nonhuman primates, various rodent species, squirrels, and pigs (Chauhan et al., 2023).

Since 1991, *Orthopoxvirus vaccinia* [vaccinia virus (VACV)] has been a persistent health concern in Brazil, with outbreaks reported in dairy cattle and humans in rural areas (Damaso et al., 2000; Domingos et al., 2021; Luques et al., 2023). VACV has also been detected in asymptomatic dogs and cats, although the epidemiological role of these animals remains uncertain (Costa et al., 2017, 2018).

Recently, a new poxvirus, the Brazilian porcupinepox virus (BPoPV), was identified as the cause of fatal cutaneous and systemic disease in wild porcupines (Hora et al., 2021). Our ongoing research indicates that this virus is widely distributed across the country, affecting both rural and urban areas. However, aside from porcupines, other potential hosts remain unknown.

The medical significance of certain poxviruses, coupled with the close contact between humans and their pets, highlights critical gaps in the scientific literature regarding the role of companion animals as potential hosts of poxviruses. Given these factors, this study aimed to assess the molecular occurrence of poxviruses in dogs and cats.

We analyzed DNA samples submitted to a reference laboratory for infectious disease screening in sick animals or those suspected of harboring infectious pathogens. The research was conducted at the Laboratório de Investigação Etiológica Veterinária (LIvE Vet) at the Universidade Federal de Uberlândia, Minas Gerais, Brazil. Uberlândia, the second most populous city in Minas Gerais, has approximately 700,000 inhabitants and serves as a hotspot for zoonotic diseases. Additionally, the city is located 542 kilometers from Belo Horizonte, the state capital, and includes both urban and rural areas. Its large urban center is surrounded by dairy farms, making it an optimal setting for investigating *Orthopoxvirus* infections, such as VACV.

Samples were collected from dogs between May and December 2022 and from cats between March 2021 and February 2023. The study included 608 dogs and 271 cats. The animals’ ages varied, ranging from 6 days to 17 years and 6 months for dogs and from 17 days to 17 years and 9 months for cats (Table 1). 

**Table 1:** Distribution of dogs and cats in each life stage.

	Age	Total (n)	Percentage (%)
Dogs	Puppy (≤1 year)	76	12.5
	Young adult (1–6 years)	264	43.4
	Mature adult (7–10 years)	143	23.5
	Senior (>10 years)	112	18.4
	Unknown	13	2.1
Total		608	100
Cats	Kitten (≤1 year)	115	42.4
	Young adult (1–6 years)	132	48.7
	Mature adult (7–10 years)	19	7.0
	Senior (>10 years)	5	1.9
Total		271	100

Cats diagnosed with immunosuppressive retrovirus infections via polymerase chain reaction (PCR) assays were also included in the study. Cats infected with *Lentivirus felimdef* [feline immunodeficiency virus (FIV)] and *Gammaretrovirus felleu* [feline leukemia virus (FeLV)]) are more susceptible to secondary infections because of the immunosuppression caused by these viruses (Hartmann, 2011). The studied population exhibited a high prevalence of FeLV (Table 2), which may increase the likelihood of detecting poxvirus infections in these cats.

**Table 2:** Classification of cats according to retroviral status.

**Retroviral status**	**Total (n)**	**Percentage (%)**
FIV	6	2.2
FeLV	64	23.6
FIV and FeLV	3	1.1
Negative	198	73.1
Total	271	100

FIV, feline immunodeficiency virus; FeLV, feline leukemia virus

Similarly, the dog population, primarily composed of individuals with clinical signs of infectious diseases and positive PCR tests for *Morbillivirus canis* [canine distemper virus (CDV)] and vector-borne pathogens such as *Ehrlichia* spp. and *Anaplasma* spp. (Table 3), reflected the trend observed in cats.

**Table 3:** Classification of dogs according to status of CDV, *Ehrlichia* spp., and *Anaplasma* spp. Infections.

**Pathogens (dogs tested, n)**	**Total (n)**	**Percentage (%)**
CDV positive (n = 105)	53	50.5
*Ehrlichia* spp. positive (n = 518)	90	17.4
*Anaplasma* spp. positive (n = 465)	65	14.0

CDV, canine distemper virus.

All DNA samples were stored at −20°C before being subjected to the poxvirus PCR assay. A real time PCR assay targeting the housekeeping gene glyceraldehyde-3-phosphate dehydrogenase (GAPDH) (Leutenegger et al., 1999) was performed to confirm DNA viability and the success of genomic DNA extraction for all samples.

All samples underwent a pan-pox PCR assay (Li et al., 2010) using 1X GoTaq^®^ Green PCR Master Mix (Promega Corporation, Madison, WI, USA), 400 nM of each primer, 3 µL of DNA sample, and nuclease-free water to a final volume of 25 µL. This assay targets a region with low GC content in poxviruses, using primers specific to the putative metalloproteinase gene. Positive control DNA from a Brazilian porcupine infected with BPoPV (GenBank MN692191) and a negative control (nuclease-free water) were included. However, no molecular evidence of any poxvirus was detected in the companion animals analyzed in this study.

In April 2022, in France, MPV DNA was detected in cutaneous lesions of a dog that lived with humans who had Mpox (Seang et al., 2022). In August 2022, a similar case was reported involving a puppy in the state of Minas Gerais, Brazil (Belo Horizonte, 2022), though no differential diagnosis was made for other dermatological conditions with similar clinical signs in dogs. Surveillance conducted on 154 dogs and cats cohabiting with humans who had confirmed Mpox in the United Kingdom found no evidence of clinical signs of MPV infection in these animals (Shepherd et al., 2022). Additionally, a recent study identified MPV DNA in 5 of 34 (14.71%) companion animals from 4 households with MPV-infected humans; although blood samples and lesions were collected from some animals, positive results were detected only in swab samples taken from the fur, ventral abdomen, oral cavity, and anorectal sites, with no serological evidence indicating active infection even during follow-up evaluations (Morgan et al., 2024). The type of sample collected could have introduced sampling bias in the present study, explaining the absence of MPV-positive results; this is because skin lesion samples are likely the most sensitive samples for detecting the virus. Furthermore, human DNA was detected in the animal samples analyzed by Morgan et al. (2024), leading the authors to conclude that there is no evidence to suggest that companion animals were infected with MPV and that the detected MPV DNA likely originated from environmental contamination from human sources. Because this study aimed to screen a large number of DNA samples already available in a biobank, it was not possible to determine whether the evaluated animals had contact with MPV-infected individuals or to perform PCR on skin samples. The data demonstrate the absence of MPV DNA in a large sample of dogs and cats, reinforcing that these animals likely do not play a significant role in transmitting or maintaining the virus in domestic settings. However, broader studies should be conducted to determine whether dogs and cats are truly refractory to MPV, particularly by investigating companion animals living with individuals affected by Mpox.

VACV infection is a significant occupational zoonosis affecting dairy workers and cattle in Brazil, as well as in other South American countries (Domingos et al., 2021). Because Minas Gerais, the state where this study was conducted, is the leading milk producer in Brazil, it represents an ideal location for identifying potential additional hosts of *Orthopoxvirus vaccinia*.

Dogs and cats from dairy farms were evaluated, with 22.81% (26/114) of dogs and 14.29% (1/7) of cats testing positive for antibodies against orthopoxviruses (Peres et al., 2013). All these samples (n = 121) were subsequently tested by PCR for VACV DNA; however, no genetic material was detected (Peres et al., 2018). In a separate study, samples from 277 urban cats in Brazil revealed that 19.13% (53/277) tested positive for anti-orthopoxvirus antibodies, while 2.17% (6/277) were positive for VACV DNA (Costa et al., 2017). Similarly, anti-orthopoxvirus antibodies were detected in 19.02% (35/184) of urban dogs from Belo Horizonte, with 20.00% (7/35) also testing positive for VACV DNA (Costa et al., 2018). These findings support the use of blood samples as a plausible method for screening VACV infection.

This study represents the first screening of companion animals in this region, and based on previous data, VACV-positive animals were expected. However, no positive cases were detected, even with a larger sample size (n = 879) than in prior studies. For future research, the ideal approach would be to combine DNA detection with antibody testing for orthopoxviruses to better understand their circulation in this population. One limitation of this study is the use of convenience samples from a molecular laboratory’s biobank, which resulted in the unavailability of serum samples for immunodiagnostic assays. Additionally, future studies should prioritize the inclusion of dogs and cats from dairy farms where vaccinia virus is present. Because of the nature of the sampling, tracing the origin of the animals in this study was not possible. However, given that the research was conducted in a large city, it is reasonable to assume that most of the animals came from an urban environment.

According to our veterinary hospital records, conflicts between wild porcupines and dogs are relatively common. BPoPV is present in the epidermis and scales of porcupines, and their quills can act as needles covered with viral particles, potentially inoculating dogs’ skin and mucous membranes during fights. Thus, the dog population in this study may have been exposed to BPoPV, although the sampling method used does not allow for tracking individual histories to determine how many dogs may have encountered porcupines. Another plausible route of exposure is the presence of wild porcupines in parks, which are also frequented by dogs. However, further studies are needed to assess the presence of BPoPV in dogs, particularly those presented for veterinary care due to porcupine quill injuries.

The significance of this study aligns with the need for more comprehensive information on poxvirus infections in companion animals. This is especially relevant given that many poxviruses are zoonotic or have zoonotic potential, alongside the increasing presence of companion animals in households worldwide.
